# Increased Dietary Inclusion Levels of Lysine Are More Effective than Arginine in Supporting the Functional Status of the Gut in Growing Turkeys

**DOI:** 10.3390/ani11082351

**Published:** 2021-08-09

**Authors:** Paweł Konieczka, Dariusz Mikulski, Katarzyna Ognik, Jerzy Juśkiewicz, Zenon Zduńczyk, Jan Jankowski

**Affiliations:** 1 Department of Poultry Science, University of Warmia and Mazury, Oczapowskiego 5, 10-718 Olsztyn, Poland; darekm@uwm.edu.pl (D.M.); janj@uwm.edu.pl (J.J.); 2 Department of Biochemistry and Toxicology, Faculty of Animal Sciences and Bioeconomy, University of Life Sciences in Lublin, Akademicka 13, 20-950 Lublin, Poland; kasiaognik@poczta.fm; 3 Institute of Animal Reproduction and Food Research of the Polish Academy of Sciences, Tuwima 10, 10-748 Olsztyn, Poland; j.juskiewicz@pan.olsztyn.pl (J.J.); z.zdunczyk@pan.olsztyn.pl (Z.Z.)

**Keywords:** lysine, arginine, gut health, microbiota activity, turkey

## Abstract

**Simple Summary:**

The concentrations of essential amino acids (EAA) in turkey diets are established in line with the recommendations of either the British United Turkeys (BUT) or the National Research Council (NRC), which, however, differ with regard to the dietary levels and ratios of important EAA, including lysine (Lys), arginine (Arg) and methionine (Met). For instance, the dietary Arg:Lys ratio in turkey diets recommended by the BUT is approximately 2–5% higher than that recommended by the NRC. Similarly, the lowest and the highest values of the Met:Lys ratio recommended by the BUT exceed those recommended by the NRC by around six and three percentage points, respectively. The above differences may appear to be relatively small, but they raise concerns in the turkey production sector due to their potential impact on both feed cost and bird physiology. Since the turkey sector has grown rapidly in the past decade, a further understanding of the EAA needs of birds is essential to achieving high growth rates and maintaining a profitable and sustainable production.

**Abstract:**

Arginine (Arg) and lysine (Lys) may be important for the overall health of turkeys. The aim of this study was to determine whether low (consistent with the guidelines) and high (10% higher than recommended) levels of dietary Arg and Lys can modulate performance and the functional status of the gut. Female turkeys were allocated to four dietary treatments (two levels of Lys (low or high) and two levels of Arg (low or high)) for a 16 wk feeding period. The treatments did not affect turkey performance determined separately for four feeding phases and for the entire 16 wk experiment (*p* > 0.05). They had no significant influence on carcass yield, meat characteristics or the associated traits either (*p* > 0.05). High-Lys diets contributed to a decrease in cecal pH, a significant increase in the concentrations of short-chain fatty acids (SCFA) and a decrease in the concentrations of putrefactive SCFA and ammonia in the cecum. High dietary levels of both amino acids significantly enhanced the activity of cecal microbiota evaluated based on extracellular enzyme activity. These findings indicate that the higher dietary level of Lys was more effective in modulating the physiological status of the gut in turkeys than Arg.

## 1. Introduction

Considerable progress in precision poultry nutrition has led to a substantial improvement in the rates of gain and feed efficiency in commercial turkey farming. The improvement has largely been driven by modifications in the nutrient composition of feed ingredients, with particular emphasis on protein and amino acids (AA). Protein and its AA composition are critical for the precise feed formulation to meet the birds’ requirements [1]. Amino acids, more specifically those that are not synthesized in the body and are, therefore, referred to as nutritionally essential AA (EAA), play a key role in the regulation of homeostasis in the whole biological system, including body maintenance, growth, immunity and reproduction [2]. Most poultry diets are based on soybean meal (SBM), which is a well-balanced protein source in terms of AA composition [3]. However, EAA, including lysine (Lys), arginine (Arg) and methionine (Met), are offered as synthetic dietary supplements.

Synthetic EAA in poultry diets offer a number of benefits, including the ability to reduce dietary protein levels, which is recommended by the nutritional guidelines for avian species. Lemme et al. [4] reported that the protein content of turkey diets could be reduced by 10% while maintaining EAA at the recommended level, without compromising bird performance up to 22 wk of age. A study by Applegate et al. [5] demonstrated that Lys, Met and threonine manipulation in turkey diets made it possible to reduce SBM levels; thus, decreasing nitrogen intake and excretion by 8.4% and 10.8%, respectively, and reducing feed cost by USD 0.37 per 20 wk-old bird. Apart from lower nitrogen emissions, precise EAA administration to birds was found to be associated with low a excretion of ammonia due to an improved protein metabolism (reviewed in [6]). The above findings indicate that the precision formulation of AA levels in poultry diets delivers both economic and environmental benefits. However, more recent reports have shown that EAA formulation may be of key importance for early nutrient availability, and that access to EAA could play a significant role in the development of the immune system in birds [7]. This is due to the fact that newly hatched poults are marginally supplied with protein and their digestive tracts are immature, which reduces their ability to digest and absorb protein [3]. Therefore, the precision formulation of EAA levels may provide birds with adequate amounts of well-balanced protein and, consequently, improve their growth and health in the following stages of development.

In general, EAA concentrations in turkey diets are established in line with the recommendations of either the British United Turkeys (BUT) [8] or the National Research Council (NRC) [9], which, however, differ with regard to the dietary levels and ratios of important EAA, including Lys, Arg and Met. For instance, the dietary Arg:Lys ratio in turkey diets recommended by the BUT [8] is approximately 2–5% higher than that recommended by the NRC [9]. Similarly, the lowest and the highest values of the Met:Lys ratio recommended by the BUT [8] exceed those recommended by the NRC [9] by around six and three percentage points, respectively [10]. The above differences may appear to be relatively small, but they raise concerns in the turkey production sector due to their potential impact on both feed cost and bird physiology [11]. Sohel [2] reported that nutrients, including AA, can modulate the expression levels of miRNA, which is responsible for the regulation of 60% of genes involved in all body processes in animals. In view of the above, the discussed differences in the inclusion levels (and ratios) of dietary EAA can have important implications and, therefore, warrant a deeper investigation. In our previous study [12], different dietary levels and ratios of Lys, Arg and Met in turkey diets had a significant effect on both the performance and immune status of turkeys. It was found that diets with increased levels of Met (45% relative to the content of dietary Lys) and Arg (110% relative to the content of dietary Lys), as compared with the nutrient requirements of turkeys (NRC recommendations) [9], led to an increase in plasma albumin concentration and in the blood concentrations of caspase-3 and caspase-8. In another study [13], different dietary Arg:Met ratios affected the performance and immune status of growing turkeys. A higher Arg level in the diet (110% vs. 90% relative to Lys content, close to the BUT recommendations [8]) decreased the blood concentrations of proinflammatory cytokines (including tumor necrosis factor alpha and interleukin 6), and immunoglobulin Y. The cited study also revealed that the increased dietary level of Met (45% vs. 30% relative to Lys content) decreased the blood concentration of interleukin 6 in 16 wk-old turkeys. In contrast, the dietary level of Arg at 90% of Lys content was associated with a lower rate of protein nitration and compromised antioxidant system function in turkeys [14]. However, turkeys’ response to different ratios of Lys and Arg in diets with high and low Met levels was more complex. In diets with the lower Met level (relative to NRC recommendations, [9]), a decrease in Arg content (90% dietary Lys) was associated with growth depression, a significant increase in the blood concentrations of total cholesterol and uric acid, whereas an increase in Arg content to 110% Lys up-regulated the blood concentration of thyroxine T4 [10]. In *Clostridium perfringens*-challenged turkeys, increased dietary levels of Arg (110% dietary Lys) and Met (45% dietary Lys) manifested their negative effects by inducing DNA oxidation and methylation in gut tissue, whereas decreased dietary levels of Arg (90% dietary Lys) inhibited this process and had a beneficial influence on gut integrity, including the up-regulation of the mRNA expression of genes determining barrier functions [15]. A number of reports have documented the complex cross-talk between the dietary levels and ratios of EAA and the host physiology. Since the turkey sector has grown rapidly in the past decade, a further understanding of the EAA needs of birds is essential to achieving high growth rates and maintaining a profitable and sustainable production. Therefore, to further investigate turkeys’ response to different dietary levels and ratios of Arg, Met and Lys, the aim of this study is to determine whether the most effective levels of Arg and Lys (established in our previous studies) can modulate physiological processes in birds, including the performance and the functional status of the gut.

## 2. Materials and Methods

### 2.1. Experimental Design and Treatments

The experimental protocol applied in this study was approved by the Local Ethics Committee in Olsztyn (Resolution No. 82/2017), and the birds were cared for according to Directive 2010/63/EU. Female Hybrid Converter turkey poults (a total of 576 birds) were supplied by a commercial hatchery (Grelavi in Kętrzyn, Poland) at the day of hatching. The poults were randomized by weight and placed in pens (2 m × 2 m per pen with initial stock density of 4.5 birds/m^2^) bedded with wood shavings. The poults were allocated to 32 pens with 18 birds per pen and 8 replicates per each of the 4 dietary treatments (144 birds/treatment). The birds were distributed among the treatments based on their body weight (BW) so as the average values of group BW did not differ significantly between the treatments in the initial growth stage. The room conditions maintained throughout the experiment were consistent with the management recommendations for Hybrid Converter turkeys [16]. The height of the feed and drink lines was adjusted to the growth stage of turkeys. The turkeys had unrestricted access to feed and water which was available ad libitum. They were reared up to 16 wk of age (hereinafter referred to as wk of feeding or wk of the feeding period), and were fed isocaloric, wheat–SBM-based diets that differed in Lys and Arg levels. The experiment had a completely randomized 2 × 2 factorial design, with 2 dietary levels of Lys (low and high) and 2 levels of Arg (low and high), resulting in 4 treatments. Throughout the experiment, the turkeys were offered diets formulated to meet their requirements in 4 feeding phases: I—(wk 1–4); II—(wk 5–8); III—(wk 9–12); IV—(wk 13–16). For each diet in the respective feeding phases, a basal mixture was prepared and its AA composition (Table 1) was determined analytically, as described by Ognik et al. [10]. Subsequently, a portion of the basal diet was mixed with the missing quantities of synthetic Lys, Arg and Met (Table 2), i.e., L-Lys HCl, L-Arginine HCl (AJINOMOTO EUROLYSINE S.A.S, Amies, France, 780 g Lys/kg and 990 g Arg/kg) and DL-Methionine (MetAMINO^®^, Evonik Degussa GmbH, Essen, Germany, 990 g Met/kg), as described by Ognik et al. [10]. The required levels of the respective AA were achieved by adding supplementary L-Lys HCl, L-Arg HCl and DL-Met on top to the basal diet. The experimental diets were prepared in a local feed mill under the direct supervision of a representative of the Department of Poultry Science, University of Warmia and Mazury. According to the experimental procedure, each batch of feed for each of the 4 feeding periods (without supplemental AA) was mixed with the appropriate amounts of Lys, Arg and Met, which had been previously mixed with approximately 10 kg of respective basal diets using a laboratory mixer. The stock-batch was added to the total batch of respective diets, and mixed thoroughly to ensure homogenous distribution in the diet. Two dietary levels of Lys were applied: low level (L_Low_ = NRC [9]) and high level (L_high_ = NRC [9] + 10%). In diets with L_Low_, L-Lysine HCl was added to the basal diet to obtain 1.60, 1.50, 1.30 and 1.00 g of Lys per 100 g of feed in 4 successive feeding periods, according to the NRC [9] requirements. L-Arginine HCl was added to the basal diet to obtain 95% and 105% Arg relative to dietary Lys content (low and high; A_Low_ and A_High_, respectively). The level of Met in the experimental diets was maintained constant, and DL-Methionine was added to obtain 0.62, 0.59, 0.51 and 0.39 g of Met per 100 g of feed in 4 successive feeding periods. As a result, the Met:Lys ratio in the L_Low_ and L_High_ diets was 0.39 and 0.35, respectively, regardless of the rearing period. The compound feed was offered as crumbles in feeding phase I, and as pellets thereafter.

### 2.2. Parameters Analyzed

The following parameters were analyzed in this study: turkey performance, carcass characteristics and selected indicators of the functional status of the gut.

#### 2.2.1. Performance Analysis

The following performance parameters were analyzed: daily body weight gain (DBWG), daily feed intake (DFI) and the feed conversion ratio (FCR). The values of BW, FI and BWG of birds were recorded on a pen basis and were used to calculate the FCR (adjusted for mortality). The performance parameters were determined separately for the 4 feeding phases (wk 1–4, 5–8, 9–12 and 13–16) and for the entire 16 wk experiment.

#### 2.2.2. Analysis of Carcass Characteristics

The following carcass characteristics and the associated traits were analyzed in 16 wk-old birds, according to the procedure described previously [18]: carcass weight, the weight of edible giblets (liver, gizzard and heart), muscles (breast, thigh, drumstick and total) and the weight of abdominal fat, relative to carcass weight. The pH of breast meat was measured 24 h after carcass chilling, using a lance pH meter (Testo GmbH 206-pH 2 m, Lenzkirch, Germany). The CIE LAB (L*a*b*) system (color space), also referred to as L*a*b* was used. Hunter L* (lightness), a* (redness) and b* (yellowness) color values were determined with the MiniScan XE Plus color difference meter (Hunter Associates Laboratory, Inc., Reston, VA, USA) in meat samples free from color defects, bruising and hemorrhages. Protein concentration was determined in the same samples of breast meat according to AOAC [19] method (*n* × 6.25; Method 990.03) and ISO Standard 1871:2009 (ISO 1871, 2009; Kjeldahl method using the automatic Kjeldahl analyzer (FOSS Tecator^TM^ 1035, Hillerød, Denmark)) [20].

#### 2.2.3. Functional Status of the Gut

At 16 wk of age, 8 turkeys representing average group BW were selected from each dietary treatment and were sacrificed after electrical stunning. The small intestine and ceca were removed and digesta samples were collected. In the jejunum, 2 cm segments were collected from the middle part; the segments were flushed with saline and placed in a 4% formaldehyde solution. Fixed dehydrated samples were embedded in paraffin. Duplicate sections of each sample were cut on a microtome, stained with hematoxylin–eosin and evaluated under a light microscope for villus height and crypt depth. The jejunal samples were additionally weighed with digesta and (after digesta sampling) the tissues were flushed with water, dried on filter paper and weighed again. Immediately after digesta collection from the small intestine, pH was measured using an electrode pH meter (pH301 pH meter, Hanna Instruments, Woonsocket, RI, USA). The dry matter (DM) content of ileal digesta was determined at 103 °C and digesta viscosity was measured using the cone/plate viscometer (model LVDV II+, Brookfield Engineering Laboratories, Stoughton, MA, USA). The weight of cecal digesta was determined, and DM, pH and ammonia concentration were measured by direct titration with sulfuric acid preceded by extraction with the use of a boric acid solution in Conway dishes. The activity of cecal microbiota was assessed based on the concentrations of short-chain fatty acids (SCFA). The concentrations of SCFA, including acetic (C2), propionic (C3), iso-butyric (C4i), butyric (C4), iso-valeric (C5i) and valeric (C5) acids as well as their profile and total pool in the ceca were determined by gas chromatography (SHIMADZU GC-2010, Kyoto, Japan) according to a previously described protocol [21]. The activity of cecal microbiota was additionally assessed based on the extracellular activity of bacterial enzymes. The extracellular activity of selected enzymes, including α- and β-glucosidase, α- and β-galactosidase, β-glucuronidase, α-arabinopyranosidase, β-xylosidase and β-cellobiosidase, was measured spectrophotometrically on a multi-plate reader (Multiskan Sky Microplate Spectrophotometer, Thermo Fisher Scientific, Waltham, MA, USA). The final concentrations of respective enzymes were calculated from the standard curves plotted for *p*-nitrophenol (PNP) or o-nitrophenol (ONP) used as standards (Sigma-Aldrich by Merck, Saint Louis, MO, USA) for the amounts of PNP or ONP liberated from the respective nitrophenyl glucoside substrates. All steps of sample processing and analytical conditions were consistent with the modified protocol of Gugołek et al. [22], described by Konieczka et al. [23].

### 2.3. Statistical Analysis

Differences between treatments were compared by two-way ANOVA as a 2 × 2 factorial design to assess the effects of 2 dietary levels of Lys and 2 dietary levels of Arg as well as their interactions. For the calculation of performance parameters, a pen was considered a replicate. The model assumptions of normality and homogeneity of variance were checked by the Shapiro–Wilk and Levene’s tests, respectively. The statistical significance of the main factors was established based on the F-test and when a significant interaction (Lys × Arg) was confirmed, the post hoc Tukey’s test was applied for a comparison of means. The differences between factors were considered significant using 95% confidence limits (*p*  <  0.05), and the variability of data was expressed as mean values with a pooled standard error (SEM). Statistical calculations were performed using the Statistica data analysis software system, version 13; Kraków, Poland [24].

## 3. Results and Discussion

### 3.1. Bird Performance, Carcass Characteristics and the Associated Traits

The administration of diets differing in Lys and Arg levels had no significant effect on turkey performance in each of the four feeding phases or during the entire experimental period (wk 1–16). The only exception was DFI in wk 5–8, which was higher (*p* = 0.032) in birds fed high-Arg diets. In the same feeding phase, DBWG increased by 3.3% (*p* = 0.057) in birds fed high-Arg diets. However, the FCR was not significantly affected by the experimental factors in wk 5–8 (Table A1). Similarly, to performance parameters, carcass characteristics (Table A2), including carcass weight, the relative weights of edible giblets, muscles and abdominal fat, and the physicochemical properties of breast meat at 16 wk of age (Table A3), did not differ significantly in turkeys receiving diets with different Lys and Arg levels (*p* > 0.05). Different results were reported by Oso et al. [25] who demonstrated that high-Arg turkey diets (where the Arg inclusion rate was around 12 percentage points higher than in the high-Arg diet in the present study) were associated with higher BW, higher carcass weight and improved dressing percentage at 112 days of age. Veldkamp et al. [26] also noted significantly higher FI at 56 days of age and higher BWG at 98 days of age in turkeys fed diets with a high Arg:Lys ratio (1.1 vs. 1.32 in the present study), whereas the FCR was not affected during the entire feeding period. The authors concluded that the Arg effect could be particularly manifested when the Lys level in the diet was marginal relative to the recommendations. In another study, the cited authors reported that increased dietary Lys levels (120% relative to NRC recommendations) affected FI and the BWG of turkeys throughout the feeding period, whereas the FCR was influenced by the experimental factors only during the first 4 wk [27]. The beneficial effect of Arg on bird performance could be attributed to its role in promoting the synthesis of protein and other molecules important for growth and tissue development (such as creatine, proline, ornithine and polyamine) and in preventing somatostatin secretion from the hypothalamus; thus, promoting growth hormone release [28,29]. Since Lys and Arg are structurally related AA, the most pronounced effect can be observed when diets with their opposite levels are fed to birds. Kidd and Kerr [30] observed a positive influence of increasing dietary Arg:Lys ratios (1.22 vs. 1.32 in the present study) on the BW and BWG of turkeys at 20 wk of age and in wk 8–20, respectively. Our previous study [14] also revealed that an increased dietary Arg content (110% of that recommended by the NRC [9], which was around 0.02 percentage points lower than that in the referred study) did not compromise turkey performance at any stage of growth or carcass characteristics at 16 wk of age when the dietary level of Lys was maintained constant, which could alleviate EAA antagonism. Nevertheless, the present study revealed that any surplus supplementation of turkey diets with either Lys or Arg was not economically justified since it neither improved performance nor reduced FI. Amino acids, next to energy, are the major contributors to feed cost. Therefore, they must be supplied in sufficient amounts, but as low as possible to meet production efficiency criteria.

### 3.2. Functional Status of the Gut

The parameters of small intestinal and cecal physiology in turkeys at 16 wk of age are presented in Table 3. The parameters of small intestinal physiology, including the weight and morphometry of the small intestine, digesta DM content, viscosity and pH, did not differ significantly (*p* > 0.05) between the treatments. However, differences were found in the parameters of cecal physiology. High-Lys diets increased the bulk of digesta (*p* = 0.033) and decreased digesta pH (*p* = 0.012). A significant interaction (Lys × Arg; *p* = 0.044) was noted for the ammonia concentration in the cecal digesta. Ammonia concentration was lowest in turkeys fed high-Lys and high-Arg diets, and high-Lys and low-Arg diets. In turkeys receiving diets with low levels of both AA and high-Arg and low-Lys diets, ammonia concentration was significantly higher than in the treatment with high Lys and Arg levels.

The physiological status of the gut is largely determined by the condition of the intestinal mucosa, which is the first structure exposed to contact with dietary compounds. Therefore, mucosal morphostructures and their parameters, such as the villus height and crypt depth, are indicators of intestinal condition [31]. The viscosity, pH and water content of ileal digesta affect the physiological status of the gut and, in consequence, can exert an indirect influence on the conditions in poultry houses. In the present study, the analyzed parameters of the small intestine were not affected by the applied dietary treatments. In contrast, dietary Lys and Arg levels had a significant effect on the cecal digesta. Particular attention should be paid to the lower pH of cecal digesta in turkeys fed high-Lys diets. The lower pH of digesta is associated with lower susceptibility to *E. coli* and *Salmonella* proliferation [32]. It appears that Lys can indirectly decrease the digesta pH by promoting the proliferation of bacteria beneficial to the host. For instance, bacteria of the genus *Lactobacillus* are known to lower the pH of the gut environment [33]. In a study by Shazali et al. [34], diets with a low crude protein content (around three percentage points lower than that recommended for Cobb broiler chickens in the finisher phase), supplemented with Lys, Met and threonine, increased the abundance of fecal lactic acid bacteria in chickens. Another beneficial effect of the applied dietary treatment was a decrease in ammonia concentration in the cecal digesta, which was particularly pronounced when high-Lys diets were fed, whereas a simultaneous increase in Arg content led to an interaction. An increased supply of EAA, including Arg, reaching the cecal environment could be responsible for the increased abundance of putrefying ammonia-producing bacteria [35].

### 3.3. Activity of Cecal Microbiota

The changes in the activity of cecal microbiota were evaluated based on SCFA concentrations (Table 4) and extracellular enzyme activity (Table 5). High-Lys diets exerted the greatest effect on SCFA concentrations. The increased inclusion rate of Lys resulted in a higher C4 concentration (*p* < 0.001), lower C5i concentration (*p* < 0.001) and, in consequence, a lower total concentrations of putrefactive SCFA (*p* = 0.010); it also increased the total concentrations of SCFA and the total SCFA pool in the cecal digesta (*p* < 0.001 and *p* = 0.002, respectively). The higher level of Lys also affected the C2 concentration and the percentage of C4 in the SCFA profile (*p* = 0.009 and *p* = 0.036, respectively) which, however, was due to a significant Lys × Arg interaction (*p* = 0.005 and *p* = 0.017, respectively). In contrast, diets with different Arg levels did not induce significant changes in SCFA concentrations. The only considerable effect exerted by high-Arg diets was an increase in the C4 concentration in the cecal digesta (*p* = 0.029). Other effects, including changes in the percentages of C2 and C4 in the SCFA profile, resulted from a significant Lys × Arg interaction (*p* = 0.003 and *p* = 0.017, respectively).

In the gut of poultry, or more specifically in the ceca, SCFA are present in high concentrations due to the high rate of fermentative metabolism by indigenous microbiota with the use of substrates that escaped digestion in the upper gastrointestinal tract. Therefore, SCFA concentrations and their profile are important indicators of the functional status of the gut. A particularly important role is played by C4 as an energy source for the enterocyte growth and the proliferation of intestinal epithelial cells, regulation of mucin production, maintenance of intestinal immune function and protection of the gut environment against pathogen colonization [36]. In the current study, the increased levels of Lys and Arg exerted a clear up-regulation effect on C4 concentration in the ceca of turkeys. However, an analysis of C4 contribution to the SCFA profile revealed that Lys was more effective in promoting C4 synthesis since its share of the SCFA profile was significantly lower when both AA were used at low levels but not when they were applied in other combinations. Thus, it seems that the Lys:Arg ratio may play a significant role in the above process, and a shift from the optimal ratio may have adverse effects on microbiota activity and the physiological function of the gut. The observed effect is more evident when both AA are supplied at low levels, which has been documented in different poultry species [37,38,39]. Protein, including resistant dietary protein, microbial protein and protein that escaped digestion, synthesized by the host, may reach the ceca and be taken up by protein-fermenting bacteria. As a result, higher concentrations of branched SCFA may be produced, pointing to unfavorable conditions in the ceca. In the present study, the increased dietary level of Lys but not Arg significantly reduced C5i concentration and the total concentrations of putrefactive SCFA in the cecal digesta. This indicates that high-Lys diets contributed to a more efficient protein utilization by turkeys and did not induce the excessive bypass of protein for putrefaction, which could potentially be used by bacteria to release end products considered harmful to the host [40]. The regulatory effect of Lys in reducing substrates available for the synthesis of putrefactive SCFA could be partially explained by the fact that Lys is the most limiting AA in turkeys, and even excessive levels of dietary Lys are well tolerated in terms of SCFA production in the cecal environment. In contrast, an insufficient supply of dietary Lys, below the chickens’ requirements, was reported to induce protein degradation to satisfy muscle requirements for protein synthesis, which suggests that the regulatory system could preferentially use Lys and make it available for cecal bacteria due to its key role in protein synthesis [41]. However, it should be noted that the SCFA profile was not disrupted in terms of the contribution of major SCFA, including C2, C3 and C4, which are formed in the greatest abundance in the ceca of turkeys [42]. However, in the present study, the SCFA-associated changes in the cecal digesta were probably affected by the larger amount of digesta and its lower pH in turkeys fed high-Lys diets, which fully corresponded to the functional response of the gut, described above. In other words, an increased Lys level could indirectly affect the cecal environment by promoting C2- and C4-producing bacteria, lowering the cecal pH and, in consequence, destroying the cells of putrefying bacteria by disturbing their ability to maintain optimal pH [43,44].

The physiological response of the gut to dietary treatments can also be measured based on the glycolytic activity of the cecal microbiota. Bacteria of the order *Clostridiales* are particularly reliable indicators due to their ability to utilize complex plant-derived carbohydrates and produce C4 [45]. Additionally, an increased extracellular enzymatic activity of the microbiome improves the overall energy capture from the diet, and is determined by the types and abundances of bacterial species residing in the cecal environment [22]. In the present study, (Table 5), increased levels of Lys and Arg enhanced the extracellular activity of α-glucosidase (*p* = 0.039 and *p* < 0.001, respectively), α-arabinopyranosidase (*p* = 0.039 and *p* < 0.001, respectively), α-arabinofuranosidase (*p* = 0.005 and *p* < 0.001, respectively) and β-xylosidase (both *p* < 0.001). In addition, the high Arg level increased the extracellular activity of β-cellobiosidase (*p* < 0.001), whereas the high Lys level increased the activity of β-glucuronidase (*p* = 0.022). A Lys × Arg interaction was found for the activity of β-glucosidase (*p* = 0.041) and α-galactosidase (*p* = 0.044); in each case, the increased dietary levels of both AA were associated with the highest activity of β-glucosidase and α-galactosidase. The data on the activity of bacterial extracellular enzymes (Table 5) indicated that Arg was more effective in promoting the extracellular activity of bacterial enzymes than Lys. According to Zhang et al. [46], an increased inclusion rate of Arg (3 g/kg higher than that recommended for Arbor Acres broiler chickens by the NRC) counteracted circulating Arg deficiency in *C. perfringens*-challenged chickens, which may indicate that in our study, larger amounts of this AA were available for stimulating the glycolytic activity of bacteria in turkeys fed high-Arg diets. The increased glycolytic activity of gut microbiota may be indicative of their better adaptation for energy uptake from the available substrates. Competition with the host for the utilization of those substrates can also be excluded since bird performance was not significantly affected. This observation is consistent with the results of other studies on poultry (reviewed by Bederska-Łojewska et al. [47]), where interactions between different enzymes and intestinal physiology were noted. On the other hand, some reports have revealed that an enhanced extracellular activity of bacterial enzymes is not always beneficial. A good example is β-glucuronidase whose high release rate points to the increased proliferation of *Clostridium* and *Salmonella* as well as to the formation of toxic and carcinogenic substances from nontoxic glycosides [48]. In the current study, the increased level of dietary Lys but not Arg was responsible for unfavorable conditions in the cecal environment. It should be noted that enhanced activities of all enzymes were associated with the increased level of dietary Arg but not Lys, which could indicate that Arg was responsible for a higher rate of fiber degradation than Lys, which in turn led to the shift in SCFA formation (mainly from C2 to C4). As a result, more energy was available for the gut microbiota; thus, decreasing the production of putrefactive SCFA in the cecal environment. However, these observations warrant deeper investigation.

## 4. Conclusions

The results of this study indicate that diets with different levels of Lys and Arg had no significant influence on turkey performance, carcass yield, meat characteristics or the associated traits. However, diets with increased concentrations of both AA significantly affected the physiological status of the gut. A more beneficial effect was exerted by the high inclusion rate of Lys, which decreased the pH of the cecal digesta, promoted the synthesis of butyric acid and reduced the concentrations of putrefactive SCFA and ammonia in the ceca. AA significantly enhanced the activity of cecal microbiota evaluated based on extracellular enzyme activity. A negative shift towards increased β-glucuronidase activity was observed only when high-Lys diets were administered.

## Figures and Tables

**Table 1:** Ingredient composition and nutrient content of basal diets (g/100 g, as-fed basis) fed to turkeys over a period of 16 wk ^1^.

Item	Feeding Period, wk			
	1–4	5–8	9–12	13–16
Ingredients				
Wheat	56.454	56.576	64.037	64.472
Maize	-	-	-	10.000
Soybean meal, 48% CP ^2^	25.380	27.722	21.121	9.324
Rapeseed meal	4.566	5.000	6.000	7.000
Potato protein	5.000	-	-	-
Soybean oil	0.892	3.088	4.725	4.371
Maize gluten meal	3.000	2.769	-	2.000
Sodium bicarbonate	0.200	0.200	0.100	0.200
Sodium chloride	0.158	0.161	0.217	0.095
Limestone	2.046	1.766	1.391	1.075
Monocalcium phosphate	1.707	1.390	1.409	0.558
L-lysine HCl	-	0.317	0.347	0.300
DL-methionine	0.195	0.231	0.163	0.072
L-threonine	0.052	0.131	0.139	0.182
Choline chloride	0.100	0.100	0.100	0.100
Vitamin–mineral premix ^3^	0.250	0.250	0.250	0.250
Titanium oxide	-	0.300	-	-
Calculated nutrient content ^4^				
Metabolizable energy, kcal/kg	2800	2900	3000	3150
Crude protein	26.35 (26.79)	24.0 (24.33)	20.3 (20.31)	17.0 (17.35)
Arginine	1.52 (1.50)	1.42 (1.38)	1.21 (1.25)	0.92 (0.89)
Lysine	1.30 (1.23)	1.35 (1.36)	1.20 (1.18)	0.90 (0.76)
Methionine	0.62 (0.61)	0.59 (0.56)	0.46 (0.45)	0.35 (0.38)
Methionine + Cysteine	1.09 (1.06)	1.03 (0.97)	0.85 (0.83)	0.71 (0.72)
Threonine	1.05 (1.03)	0.97 (0.94)	0.84 (0.83)	0.76 (0.78)
Calcium	1.25	1.10	0.95	0.65
Available phosphorus	0.65	0.55	0.47	0.32

^1^ Source: Ognik et al. [17]. ^2^ 480 g/kg crude protein. ^3^ Provided per kg diet (in the respective feeding periods (wk): 0–4, 5–8, 9–12 and 13–16): mg: retinol 3.78, 3.38, 2.88 and 2.52; cholecalciferol 0.13, 0.12, 0.10 and 0.09; a-tocopheryl acetate 100, 90, 80 and 70; vit. K3 5.8, 5.6, 4.8 and 4.2; thiamine 5.4, 4.7, 4.0 and 3.5; riboflavin 8.4, 7.5, 6.4 and 5.6; pyridoxine 6.4, 5.6, 4.8 and 4.2; cobalamin 0.032, 0.028, 0.024 and 0.021; biotin 0.32, 0.28, 0.24 and 0.21; pantothenic acid 28, 24, 20 and 18; nicotinic acid 84, 75, 64 and 56; folic acid 3.2, 2.8, 2.4 and 2.1; Fe 64, 60, 56 and 48; Mn 120, 112, 96 and 84; Zn 110, 103, 88 and 77; Cu 23, 19, 16 and 14; I 3.2, 2.8, 2.4 and 2.1; Se 0.30, 0.28, 0.24 and 0.21, respectively. ^4^ The value in parentheses was determined analytically.

**Table 2:** Amounts of supplemental synthetic amino acids added to the basal diet, g/100 g ^1^.

Item	Treatment ^2^			
	L_Low_A_Low_	L_Low_A_High_	L_High_A_Low_	L_High_A_High_
1–4 wk				
L-lysine HCl	0.47	0.47	0.67	0.67
L-arginine HCl	0.02	0.18	0.17	0.35
DL-methionine	0	0	0	0
5–8 wk				
L-lysine HCl	0.18	0.18	0.37	0.37
L-arginine HCl	0.05	0.20	0.19	0.35
DL-methionine	0.03	0.03	0.03	0.03
9–12 wk				
L-lysine HCl	0.15	0.15	0.32	0.32
L-arginine HCl	0	0.12	0.11	0.25
DL-methionine	0.06	0.06	0.06	0.06
13–16 wk				
L-lysine HCl	0.30	0.30	0.43	0.43
L-arginine HCl	0.06	0.16	0.16	0.27
DL-methionine	0.01	0.01	0.01	0.01

L-lysine HCl contained 780 g lysine/kg (AJINOMOTO EUROLYSINE S.A.S, Amies, France). L-arginine HCl contained 990 g arginine/kg (AJINOMOTO EUROLYSINE S.A.S). DL-methionine contained 990 g methionine/kg (MetAMINO^®^; Evonik Degussa GmbH, Essen, Germany). ^1^ Source: Ognik et al. [17]. ^2^ L_Low_A_Low_, diet with low Lys and low Arg levels; L_Low_A_High_, diet with low Lys and high Arg levels; L_High_A_Low_, diet with high Lys and low Arg levels; L_High_A_High_, diet with high Lys and high Arg levels.

**Table 3:** Parameters of small intestinal and cecal physiology in experimental turkeys.

Item				Small Intestine				Ceca				
	Villus Height, µm	Crypt Depth, µm	Villus Height/Crypt Depth	Mass with Digesta	Viscosity, Ileal	Dry Matter, Ileal	pH, Ileal	Tissue	Digesta	Dry Matter	NH_3_	pH
				g/kg BW	mPa s	%		g/kg BW	g/kg BW	%	mg/g	
Group (*n* = 8) ^1^												
L_Low_A_Low_	1264.9	134.3	9.43	28.0	2.46	17.2	6.26	3.34	3.46	13.8	0.713 ^a^	6.52
L_Low_A_High_	1271.8	137.8	9.24	27.7	2.29	17.5	6.20	3.35	3.11	14.6	0.771 ^a^	6.56
L_High_A_Low_	1246.4	133.0	9.38	26.8	2.53	17.4	6.28	3.33	4.08	13.5	0.662 ^ab^	6.33
L_High_A_High_	1267.6	135.0	9.40	26.9	2.49	16.7	6.13	3.64	4.31	14.9	0.560 ^b^	6.10
SEM	8.59	0.97	0.081	0.620	0.097	0.268	0.059	0.098	0.211	0.330	0.023	0.066
Lysine (Lys)												
Low	1268.3	136.0	9.34	27.9	2.37	17.4	6.23	3.34	3.28 ^b^	14.2	0.742	6.54 ^a^
High	1257.0	134.0	9.39	26.8	2.51	17.0	6.21	3.49	4.19 ^a^	14.2	0.611	6.21 ^b^
SEM												
Arginine (Arg)												
Low	1255.6	133.6	9.41	27.4	2.50	17.3	6.27	3.33	3.77	13.7	0.688	6.42
High	1269.7	136.4	9.32	27.3	2.39	17.1	6.17	3.49	3.71	14.8	0.666	6.33
SEM	48.85	5.35	0.465									
*p* value												
Lys	0.528	0.307	0.751	0.441	0.490	0.577	0.835	0.482	0.033	0.998	<0.001	0.012
Arg	0.434	0.163	0.615	0.942	0.604	0.731	0.405	0.435	0.885	0.108	0.571	0.461
Lys × Arg	0.688	0.699	0.555	0.828	0.742	0.386	0.749	0.479	0.484	0.645	0.044	0.277

^1^ L_Low_A_Low_, diet with low Lys and low Arg levels; L_Low_A_High_, diet with low Lys and high Arg levels; L_High_A_Low_, diet with high Lys and low Arg levels; L_High_A_High_, diet with high Lys and high Arg levels. ^a,b^ Mean values within a column and in groups followed by different superscript letters are significantly different (*p* < 0.05); the differences among the groups were indicated with superscripts only when a significant Lys × Arg interaction was noted (*p* < 0.05); when a significant Lys × Arg interaction was noted, the differences within the main effects (Lys or Arg) were not shown. SEM—standard error of the mean (SD divided by the square root of replication number, *n* = 16).

**Table 4:** Concentrations of short-chain fatty acids (SCFA) in the cecal digesta of experimental turkeys.

	Concentration, μmol/g								SCFA Profile, %			SCFA Pool
	C2	C3	C4i	C4	C5i	C5	PSCFA	Total SCFA	C2	C3	C4	
Group (*n* = 8) ^1^												
L_Low_A_Low_	48.5 ^a^	5.17	0.472	12.6	0.699	0.588	1.76	68.1	71.3 ^a^	7.63	18.4 ^b^	236
L_Low_A_High_	39.9 ^b^	5.23	0.385	16.5	0.679	0.764	1.83	63.5	62.8 ^b^	8.13	26.2 ^a^	199
L_High_A_Low_	47.9 ^a^	5.27	0.308	18.6	0.410	0.810	1.53	73.3	65.4 ^b^	7.06	25.4 ^a^	298
L_High_A_High_	53.2 ^a^	5.87	0.245	20.7	0.288	0.664	1.20	80.9	65.7 ^b^	7.16	25.7 ^a^	350
SEM	1.380	0.460	0.039	0.806	0.047	0.045	0.086	1.909	0.855	0.587	0.904	18.2
Lysine (Lys)												
Low	44.2	5.20	0.428	14.6 ^b^	0.689 ^a^	0.676	1.79 ^a^	65.8 ^b^	67.1	7.88	22.3	217 ^b^
High	50.5	5.57	0.276	19.6 ^a^	0.349 ^b^	0.737	1.36 ^b^	77.1 ^a^	65.6	7.11	25.5	323 ^a^
Arginine (Arg)												
Low	48.2	5.22	0.390	15.6 ^b^	0.555	0.699	1.64	70.7	68.4	7.34	21.9	267
High	46.6	5.55	0.315	18.6 ^a^	0.484	0.714	1.51	72.2	64.3	7.64	25.9	274
*p* value												
Lys	0.009	0.704	0.052	<0.001	<0.001	0.500	0.010	<0.001	0.275	0.535	0.036	0.002
Arg	0.473	0.734	0.325	0.029	0.349	0.867	0.408	0.646	0.005	0.805	0.012	0.819
Lys × Arg	0.005	0.776	0.876	0.475	0.500	0.083	0.210	0.066	0.003	0.872	0.017	0.168

C2: acetate; C3: propionate; C4i: isobutyrate; C4: butyrate; C5i: isovalerate; C5: valerate; PSCFA: putrefactive SCFA (sum of iso-butyric, iso-valeric and valeric acids); SCFA pool: total pool of SCFA expressed in μmol/kg body weight. ^1^ L_Low_A_Low_, diet with low Lys and low Arg levels; L_Low_A_High_, diet with low Lys and high Arg levels; L_High_A_Low_, diet with high Lys and low Arg levels; L_High_A_High_, diet with high Lys and high Arg levels. ^a,b^ Mean values within a column and in groups followed by different superscript letters are significantly different (*p* < 0.05); the differences among the groups were indicated with superscripts only when a significant Lys × Arg interaction was noted (*p* < 0.05); when a significant Lys × Arg interaction was noted, the differences within the main effects (Lys or Arg) were not shown. SEM—standard error of the mean (SD divided by the square root of replication number, *n* = 32); interaction between two main effects (dietary levels of Lys and Arg).

**Table 5:** Activity of bacterial extracellular enzymes (µmol/h/g) in the cecal digesta of experimental turkeys.

	Glucosidase		Galactosidase		β-Glucuronidase	α-Arabinopyranosidase	α-Arabinofuranosidase	β-Xylosidase	β-Cellobiosidase
	α-	β-	α-	β-					
Group (*n* = 8) ^1^									
L_Low_A_Low_	27.2	7.09 ^b^	60.5 ^b^	35.2	61.8	3.30	2.52	6.13	0.143
L_Low_A_High_	34.4	8.59 ^b^	72.4 ^b^	57.4	43.1	4.66	5.00	9.52	0.199
L_High_A_Low_	29.2	8.78 ^b^	58.9 ^b^	26.1	67.5	3.69	3.18	8.66	0.156
L_High_A_High_	48.7	17.7 ^a^	104.0 ^a^	44.9	66.0	6.75	6.19	16.6	0.257
SEM	2.333	1.116	4.917	3.430	3.310	0.363	0.300	0.954	0.012
Lysine (Lys)									
Low	30.8 ^b^	7.83	66.5	46.3	52.4 ^b^	3.98 ^b^	3.76 ^b^	7.82 ^b^	0.171
High	38.9 ^a^	13.2	81.3	35.5	66.7 ^a^	5.22 ^a^	4.68 ^a^	12.6 ^a^	0.207
Arginine (Arg)									
Low	28.2 ^b^	7.93	59.7	30.7 ^b^	64.7	3.49 ^b^	2.85 ^b^	7.39 ^b^	0.149 ^b^
High	41.6 ^a^	13.2	88.0	51.1 ^a^	54.5	5.71 ^a^	5.60 ^a^	13.1 ^a^	0.228 ^a^
*p* value									
Lys	0.039	0.004	0.067	0.069	0.022	0.039	0.005	<0.001	0.067
Arg	<0.001	0.005	<0.001	<0.001	0.096	<0.001	<0.001	<0.001	<0.001
Lys × Arg ^2^	0.111	0.041	0.044	0.770	0.157	0.147	0.398	0.104	0.236

^1^ L_Low_A_Low_, diet with low Lys and low Arg levels; L_Low_A_High_, diet with low Lys and high Arg levels; L_High_A_Low_, diet with high Lys and low Arg levels; L_High_A_High_, diet with high Lys and high Arg levels. ^a,b^ Mean values within a column and in groups followed by different superscript letters are significantly different (*p* < 0.05); the differences among the groups were indicated with superscripts only when a significant Lys × Arg interaction was noted (*p* < 0.05); when a significant Lys × Arg interaction was noted, the differences within the main effects (Lys or Arg) were not shown. SEM—standard error of the mean (SD divided by the square root of replication number, *n* = 32); ^2^ interaction between two main effects (dietary levels of Lys and Arg).

## Data Availability

The datasets used and/or analyzed during the current study are available from the corresponding authors on reasonable request.

## Appendix A

**Table A1:** Body weight gain, daily feed intake and feed conversion ratio of turkeys fed diets differing in Lys and Arg content.

Item	1–4 wk			5–8 wk			9–12 wk			13–16 wk			1–16 wk		
	DBWG (g)	DFI (g)	FCR (g/g)	DBWG (g)	DFI (g)	FCR (g/g)	DBWG (g)	DFI (g)	FCR (g/g)	DBWG (g)	DFI (g)	FCR (g/g)	DBWG (g)	DFI (g)	FCR (g/g)
Treatment ^1^															
L_Low_A_Low_	37.9	52.7	1.39	108.1	185.9	1.72	151.1	362.0	2.40	116.4	410.0	3.53	99.3	235.3	2.37
L_Low_A_High_	38.4	53.6	1.40	110.3	188.3	1.71	151.5	361.2	2.39	112.7	411.9	3.67	99.1	235.5	2.38
L_High_A_Low_	37.3	51.8	1.39	106.8	181.5	1.70	152.6	357.6	2.35	116.8	415.5	3.57	98.7	233.0	2.36
L_High_A_High_	38.0	52.7	1.39	111.9	191.8	1.71	150.4	361.5	2.41	112.7	406.6	3.61	99.5	236.8	2.38
SEM	0.245	0.36	0.004	0.934	1.510	0.007	1.060	1.821	0.011	1.302	2.553	0.031	0.471	1.188	0.008
Lysine (Lys)															
Low	38.2	53.2	1.39	109.2	187.1	1.72	151.3	361.6	2.39	114.6	411.0	3.60	99.2	235.4	2.37
High	37.7	52.3	1.39	109.4	186.6	1.71	151.5	359.5	2.38	114.7	411.0	3.59	99.1	234.9	2.37
Arginine (Arg)															
Low	37.6	52.3	1.39	107.5	183.7 ^b^	1.71	151.8	359.8	2.37	116.6	412.7	3.55	99.0	234.1	2.37
High	38.2	53.2	1.39	111.1	190.1 ^a^	1.71	150.9	361.4	2.40	112.7	409.2	3.64	99.3	236.1	2.38
*p* value															
Lys	0.317	0.216	0.496	0.917	0.865	0.589	0.937	0.580	0.496	0.948	0.989	0.893	0.921	0.844	0.854
Arg	0.238	0.226	0.805	0.057	0.032	0.884	0.688	0.678	0.280	0.152	0.509	0.157	0.764	0.422	0.460
Lys×Arg	0.877	0.958	0.819	0.433	0.173	0.378	0.573	0.538	0.127	0.954	0.309	0.488	0.660	0.474	0.666

DBWG, daily body weight gain; DFI, daily feed intake; FCR, feed conversion ratio; ^1^ L_Low_A_Low_, diet with low Lys and low Arg levels; L_Low_A_High_, diet with low Lys and high Arg levels; L_High_A_Low_, diet with high Lys and low Arg levels; L_High_A_High_, diet with high Lys and high Arg levels. ^a,b^ values in same column with no common superscripts are significantly different (*p* < 0.05). Data are means of 8 replicates (*n* = 8).

**Table A2:** The effect of diets differing in Lys and Arg content on carcass characteristics in turkeys at 16 weeks of age (g·100 g^–1^ body weight).

	BW before Slaughter, kg	Carcass	Edible Giblets			Muscle				Abdominal Fat
			Liver	Gizzard	Heart	Breast	Thigh	Drumstick	Total Muscle	
Treatment ^1^										
L_Low_A_Low_	11.32	81.51	1.29	0.51	0.32	21.18	10.55	7.71	39.45	1.71
L_Low_A_High_	11.30	81.68	1.35	0.49	0.31	21.11	10.80	8.02	39.92	1.76
L_High_A_Low_	11.39	81.68	1.48	0.49	0.29	21.24	10.55	8.18	39.97	2.02
L_High_A_High_	11.39	81.82	1.32	0.44	0.32	21.57	10.36	7.89	39.81	1.91
SEM	0.057	0.199	0.039	0.012	0.005	0.183	0.104	0.122	0.271	0.069
Lysine (Lys)										
Low	11.31	81.60	1.32	0.50	0.32	21.15	10.67	7.86	39.68	1.73
High	11.39	81.75	1.40	0.47	0.31	21.40	10.45	8.03	39.89	1.96
Arginine (Arg)										
Low	11.35	81.60	1.39	0.50	0.31	21.21	10.55	7.94	39.71	1.86
High	11.35	81.75	1.33	0.47	0.31	21.34	10.58	7.95	39.87	1.83
*p* value										
Lys	0.501	0.716	0.328	0.136	0.325	0.500	0.304	0.501	0.717	0.105
Arg	0.959	0.717	0.472	0.177	0.345	0.742	0.917	0.970	0.783	0.819
Lys × Arg	0.917	0.972	0.163	0.643	0.131	0.604	0.312	0.245	0.582	0.565

^1^ L_Low_A_Low_, diet with low Lys and low Arg levels; L_Low_A_High_, diet with low Lys and high Arg levels; L_High_A_Low_, diet with high Lys and low Arg levels; L_High_A_High_, diet with high Lys and high Arg levels. Data are means of 8 replicates (*n* = 8).

**Table A3:** The effect of diets differing in Lys and Arg content on the physicochemical properties of breast meat in turkeys at 16 weeks of age.

Item	pH_24_	Color			Protein, %
		Lightness, L*	Redness, a*	Yellowness, b*	
Treatment ^1^					
L_Low_A_Low_	5.75	56.28	4.25	10.93	25.34
L_Low_A_High_	5.72	56.05	3.74	11.33	25.13
L_High_A_Low_	5.58	55.23	4.35	11.59	25.34
L_High_A_High_	5.70	54.92	4.14	11.42	25.20
SEM	0.041	0.419	0.121	0.163	0.071
Lysine (Lys)					
Low	5.73	56.16	4.00	11.13	25.23
High	5.64	55.08	4.24	11.51	25.27
Arginine (Arg)					
Low	5.66	55.76	4.30	11.26	25.34
High	5.71	55.48	3.94	11.38	25.16
*p* value					
Lys	0.266	0.216	0.310	0.260	0.823
Arg	0.562	0.754	0.143	0.724	0.231
Lys × Arg	0.365	0.961	0.522	0.389	0.792

pH_24_: pH measured after 24 h The CIE LAB (L*a*b*) system (color space) was used for Hunter L* (lightness), a* (redness) and b* (yellowness) color values determination. ^1^ L_Low_A_Low_, diet with low Lys and low Arg levels; L_Low_A_High_, diet with low Lys and high Arg levels; L_High_A_Low_, diet with high Lys and low Arg levels; L_High_A_High_, diet with high Lys and high Arg levels. Data are means of 8 replicates (*n* = 8).
